# Home improvement and system-based health promotion for sustainable prevention of Chagas disease: A qualitative study

**DOI:** 10.1371/journal.pntd.0007472

**Published:** 2019-06-13

**Authors:** Claudia Nieto-Sanchez, Benjamin R. Bates, Darwin Guerrero, Sylvia Jimenez, Esteban G. Baus, Koen Peeters Grietens, Mario J. Grijalva

**Affiliations:** 1 Centro de Investigación para la Salud en América Latina, Escuela de Ciencias Biológicas, Facultad de Ciencias Exactas y Naturales, Pontificia Universidad Católica del Ecuador, Quito, Ecuador; 2 Infectious and Tropical Disease Institute, Department of Biomedical Sciences, Heritage College of Osteopathic Medicine, Ohio University, Athens, Ohio, United States of America; 3 Medical Anthropology Unit, Department of Public Health, Institute of Tropical Medicine, Antwerp, Belgium; 4 School of Communication Studies, Ohio University, Athens, Ohio, United States of America; 5 Facultad de Arquitectura, Arte y Diseño, Pontificia Universidad Católica del Ecuador, Quito, Ecuador; Instituto de Ciências Biológicas, Universidade Federal de Minas Gerais, BRAZIL

## Abstract

**Background:**

Human transmission of Chagas disease (CD) most commonly occurs in domiciliary spaces where triatomines remain hidden to feed on blood sources during inhabitants’ sleep. Similar to other neglected tropical diseases (NTDs), sustainable control of CD requires attention to the structural conditions of life of populations at risk, in this case, the conditions of their living environments. Considering socio-cultural and political dynamics involved in dwellings’ construction, this study aimed to explore social factors that contribute or limit sustainability of CD’s prevention models focused on home improvement.

**Methods and main findings:**

Using Healthy Homes for Healthy Living (HHHL)—a health promotion strategy focused on improvement of living environments and system-based health promotion—as a reference, a qualitative study was conducted. Research participants were selected from three rural communities of a CD endemic region in southern Ecuador involved in HHHL’s refurbishment and reconstruction interventions between 2013 and 2016. Folowing an ethnographic approach, data were collected through interviews, participant observation, informal conversations and document analysis. Our results indicate that the HHHL model addressed risk factors for CD at the household level, while simultaneously promoting wellbeing at emotional, economic and social levels in local communities. We argue that sustainability of the CD prevention model proposed by HHHL is enhanced by the confluence of three factors: systemic improvement of families’ quality of life, perceived usefulness of control measures, and flexibility to adapt to emerging dynamics of the context.

**Conclusion:**

HHHL’s proposed home improvement, facilitated through system-based rather than disease specific health promotion processes, enhances agency in populations at risk and facilitates community partnerships forged around CD prevention. Although an independent analysis of cost-effectiveness is recommended, structural poverty experienced by local families is still the most important factor to consider when evaluating the sustainability and scalability of this model.

## Author summary

Chagas disease (CD) is transmitted by triatomine insects, vectors of the parasite *Trypanosoma cruzi*. Triatomines are commonly found in precariously constructed homes where they remain hidden in cracks and crevices during the day and feed on blood sources at night. Due to this association between living environments and disease, multiple control programs have implemented some form of home improvement as a CD preventive measure. Using Healthy Homes for Healthy Living (HHHL)—a strategy focused on home improvement and health promotion activities conducted through community partnerships—as reference, this study was designed to explore factors affecting sustainability of such models of disease control. Research participants were selected from families that have participated in construction projects implemented by HHHL between 2013 and 2016 in three rural communities of southern Ecuador. Following qualitative methodological approaches, data were collected through interviews, participant observation, informal conversations and document analysis. Our results indicate that home improvement, when conducted under systemic approaches to disease prevention, can lead to a comprehensive idea of health expressed as individual physical protection, as well as a emotional, economic, and social wellbeing at household and community levels. Sustainability of this intervention is linked to an increased sense of agency around disease prevention in local families.

## Introduction

Chagas disease (CD) is caused by *Trypanosoma cruzi* (*T*. *cruzi*), a protozoan parasite found in the hindgut of blood-sucking insects known as triatomines. Recent estimates report around 6 to 7 million people infected with *T*. *cruzi* in Latin America, approximately 200,000 of them in Ecuador [1]. The most common route of human transmission of CD occurs in domiciliary environments where triatomines can remain hidden in cracks and crevices during the day and become active at night to search for blood sources. Once infection occurs, people can show variable symptoms or remain asymptomatic for long periods of time, until they develop the chronic phase of *T*. *cruzi* infection. At this stage, infected individuals can experience arrhythmias, palpitations, and chest pain [2]. About 30% to 40% of the affected population develops cardiopathies, alterations of the gastrointestinal system such as megacolon and megaesophagus, neurological disorders or a mix of clinical manifestations in later stages of the disease [3,4].

CD has been classified as a neglected tropical disease because it mainly affects people living in poverty—mainly in tropical and subtropical regions of the Americas. Although important progress been made in the last few years in terms of diagnostic tools and treatment [5–7], CD has historically received limited attention from researchers, medical communities and policymakers [8–10]. Ecuador, in particular, presents one of the highest percentages of population at risk of CD due to domiciliary infestation in the continent [11]. In spite of control efforts implemented by the National Chagas Disease Control Program since 2003 [11,12], CD persists in areas where environmental factors and living conditions create favorable habitats for triatomine infestation.

Ongoing contact between humans and CD vectors increases the possibilities of contracting the infection and developing the disease [13]. Current control strategies recommended by the World Health Organization (WHO) are mainly focused in interrupting CD’s transmission cycle in the intersection between vectors and humans thorough selective or community wide indoor insecticide spraying, along with information and education activities [14]. Although these standardized control measures are effective in the short term, they ignore systemic relations between community health, productive activities and environmental conditions also associated with the disease that remain intact and facilitate re-infestation once insecticides’ protective capacity subsides [13,15,16]. Different from disease-centered approaches, systemic approaches to disease prevention identify and anticipate these synergies, reactions and interactions between actors and contexts in order to increase effectiveness of interventions [17].

Consequently, multiple CD control programs have also proposed some form of home improvement to interrupt vectors’ circulation from natural to human environments and reduce harbourages for the bugs [18]. Such improvements include provision of high quality and durable plastering materials [13,19], amelioration of ventilation and illumination openings [20], wall cracks’ fixing [21], and complete replacement of dwellings [22]. Installation of zinc or iron roofs, fitting of solid floors, and improvement of peridomestic environments have also been recommended to keep CD’s vectors away from human living environments [23]. Most of these alterations have been implemented to control other vector-borne diseases and constitute an attempt to test multi-sectoral approaches that reduce public health reliance on insecticides and bring sustainable reduction of vectors’ breeding sites [24].

Home improvement is considered a sustainable form of vector control, not only because of the long-term protective effect expected from infrastructure-based interventions, but also because of the impact that housing structures can have on the overall health [23,25–28] and socio-economic conditions [22,29] of their inhabitants. However, this form of vector control is usually challenged by implementation issues—including cost-effectiveness, limited accessibility to populations at risk, low availability of construction materials, high demand of technical expertise and scalability issues [25,27,30,31]—challenging the feasibility of bringing infrastructure-based protective measures up to scale, and, as a consequence, the practicality of this measure for long-term CD control.

As a response to these conflicting views, it has been recommended that infrastructure-based interventions become more than technical processes of home reconstruction and, instead, adopt systemic and interdisciplinary approaches to address the environmental, economic, and social factors challenging their sustainability [23]. Systemic perspectives are considered when attempting to include not only the biomedical conditions leading to disease occurrence, but also the different forms of exclusion that constitute the experience of marginalization for affected populations [32–35]. Perspectives such as Ecohealth have contributed in this direction by providing concrete rationales to promote activities such as educational workshops, productive intiatives, participant-based reflective exercises, and removal and cleaning of shelters for domestic animals [21,36,37] under specific CD protective infrastructure interventions. Similarly, context-specific forms of community involvement have been added to home improvement strategies to address social and ecological variables involved in the domestic, peridomestic and sylvatic cycles of the disease [20,24,38], while participatory strategies designed under socio-ecological perspectives [39,40] have looked at increasing the sustainability of these interventions through strategies focused on individual [41], interpersonal [42], community [43] and institutional levels of impact [44].

This study was embedded in Healthy Homes for Healthy Living, HHHL [45–47]—briefly, an initiative focused on building and promoting CD protective living environments in rural homes of southern Ecuador. We aimed to identify factors that contribute or limit sustainability of CD prevention programs based on building and promoting protective living environments.

## Methods

### Study site

This study was conducted in three communities of Loja province (southern Ecuador) that have registered high triatomine infestation rates [48]: Chaquizhca (48%), Guara (36.4%) and Bellamaria (17.1%). Vegetation is characterized by bushes and herbaceous plants, while the main agricultural crops include corn, kidney beans, yucca, fuits, peanuts, and coffee. Like most people in the province, families in these villages rely on subsitance agriculture and animal husbandry as their main economic activities. Productive activities are organized around two major periods: a dry season going from mid-May through November, and a dry season extending from December to April approximately.

Practices and behaviors linked with increased risk of triatomine infestation such as cohabitation with domestic animals, accumulation of produce within the household and regular occupation of adobe structures are part of local routines [15,49]. Most homes in these communities are adobe constructions following a basic pattern of walls raised on top of stone foundations assembled above the ground, dirt floors, wooden beams, bamboo ceilings, and clay tile roofs. Under this model, walls and foundations remain visible and exposed to environmental conditions throughout the years. Structural problems increase when adobe surfaces are washed off as a result of ongoing wind and water friction. Domiciliary triatomine infestation in this province has been previously associated with the presence of pigs and goats in perdomestic areas, lack of latrine/toilet, storage of agricultural products inside the house and presence of fruit trees in the proximities of the home [48].

Although different in aspects such as proximity to urban centers or family size, the intervention communities are relatively homogeneous. Many community members in Chaquizhca and Guara are close or distant relatives, while Bellamaria’s families are mostly descendants of settlers’ movements that earned titles over occupied territories in this area after a land reform in the 1960s. Considering that the regular size of local homes is about 65 m2 and host families of four to ten members, issues derived from overcrowding are common.

### The Healthy Homes for Healthy Living model (HHHL)

Healthy Homes for Healthy Living (HHHL) is part of the Healthy Living Initiative (HLI), a multi-year research and service project designed to address socio-economic dynamics contributing to CD occurrence in Loja province [45,46,50]. Led by the Infectious and Tropical Disease Institute (ITDI) at Ohio University (OU) and the Center for Research in Health in Latin America (CISeAL) at Pontifical Catholic University of Ecuador (PUCE), the project is interested in exploring strategies for long-term CD control. In collaboration with national institutions and international NGOs such as Plan International, Ayuda en Acción and Rotary Club International, HLI facilitated the construction of drinking water systems, formalization of income generation activities, and capacity building for local leaders in communities with active Chagas disease transmission in Southern Ecuador since 2009.

HHHL proposes a strategy focused on building and promoting living environments designed to deter presence of triatomines in domestic and peridomestic areas of rural homes in this region. The HHHL’s model promotes structural improvement of homes (Table 1), health promotion activities at the household level (Table 2), and community involvement in income generation activities as main intervention lines.

**Table 1:** Anti-triatomine measures installed as part of HHHL’s infrastructure intervention.

Intervention in domiciliary areas	
**Full reconstruction**	**Rationale**
Underground foundations made out of concrete, cement columns, and steel rods.	Adding seismic resistance to the general structure.
Construction of a new home that includes a kitchen, two or three rooms (depending on the size of the family), and social area (porch).	Reducing overcrowding.
Walls made out of small adobe blocks secured by mesh and plastered with compressed earth block (CEB) and stucco. Interior walls painted in light color. Sealed space between wall and ceiling.	Securing resistance of the construction and adherence of plastering to avoid cracks and crevices that could offer habitats for triatomines. Easy identification of insects inside the home space.
Floors made out of compressed earth block (CEB).	Reducing holes in the floor where triatomines could hide.
Roofs structured with wood beams and covered by clay tiles and sheets of waterproofed asphalt.	Improving safety and ventilation. Reducing storage areas in ceilings that could become habitats for triatomines.
Mesh, glass and wood window protection.	Facilitating cross-ventilation and reducing insects’ circulation.
Wooden doors protected by mesh screens.	Reducing circulation of domestic animals and insects.
Kitchen counter and improved wood stove.	Expanding safe areas for food management and reducing circulation of smoke inside the home.
Refurbishment of sanitary facilities.	Improving management of human waste.
Demolition of existing home.	Avoiding reoccupation of triatomine-prone spaces.
**Partial improvement**	**Rationale**
Mesh, glass and wood window protection.	Facilitating cross-ventilation and reducing insects’ circulation.
Ceiling construction and roof reparation.	Improving internal temperature and reducing water leaks. Preventing insect entrance.
Plastering of holes and cracks.	Eliminating hiding spaces for triatomines.
Door mesh.	Reducing circulation of domestic animals and insects.
**Intervention in peridomestic areas (for reconstructed and refurbished homes)**	
Construction of fences.	Interrupting circulation of animals from the natural environment to domestic areas.
Construction of animals’ shelters.	Reducing permanent presence of domestic animals in domestic areas.
Construction of storage facilities.	Reducing storage needs inside and around the homes.
Organization of productive gardens.	Increasing income generation opportunities.

**Table 2:** Health promotion activities implemented by HHHL.

HHHL Health Promotion Actions	
**Pre-intervention**	• Identification of existing uses of the space • Socio-economic evaluation • Agreement on intervention plan • Introduction of health promotion practices through educational materials
**Intervention**	• Relocation of families in temporary homes • Promotion of safety measures during the construction • Mediation between partners and other actors involved in the construction process • Facilitation of administrative processes • Monitoring of health promotion practices
**Post-intervention**	• Facilitation of reoccupation process • Follow up to uses of triatomine protective practices in the new home • Training on productive use of peridomestic areas

This study was conducted during HHHL’s pilot phase, aimed at collecting information about the technical and social implications of this type of intervention in Loja province. Interventions conducted during the pilot phase were developed in three different moments (Table 3): prototyping (2013), refurbishment (2014), and simultaneous reconstruction (2015 and 2016). Four homes (4) were fully reconstructed (GA = 2, CH = 1, BM = 1) and two more were refurbished (CH = 1, BM = 1) during this phase of intervention.

**Table 3:** HHHL’s interventions during pilot phase.

Year	Village*	Pilot phase	Goal
2013	GA	Prototype–full reconstruction	- Prototyping an anti-triatomine home adapted to the physical and cultural conditions of the region. - Designing health promotion processes to facilitate appropriation of protective measures in fully reconstructed homes.
2014	CH	Refurbishment	Testing anti-triatomine measures in dwellings that did not require full reconstruction, including social housing homes built by the National Ministry of Housing (MIDUVI).
	BM		
2015 - 2016	BM	Full reconstruction	Identifying logistical and social implications of conducting differentiated and simultaneous interventions around home improvement for CD prevention.
	GA		
	CH		

*Village: GA = Guara; CH = Chaquizhca; BM = Bellamaria

During the pilot phase, HHHL worked under a model of community partnerships established on the basis of the contributions that different actors could make to the physical intervention of the space, including families, community members, and representatives of the local government. The term ‘partner family’ was adopted to refer to families that agreed to build or improve their homes according to the HHHL model. Partner families in each stage of intervention were selected according to their expressed interest in reconstructing their homes, the capacity to commit resources to the project, and the decay status of their dwellings [45]. Additional socio-economic analyses were conducted to design different partnership schemes considering factors impacting the economic situation of local families, including number of school age children, property of a productive plot, previously acquired loans, access to government subsidies, main economic activities, and recent migration to urban centers, among others.

**Fig 1:**
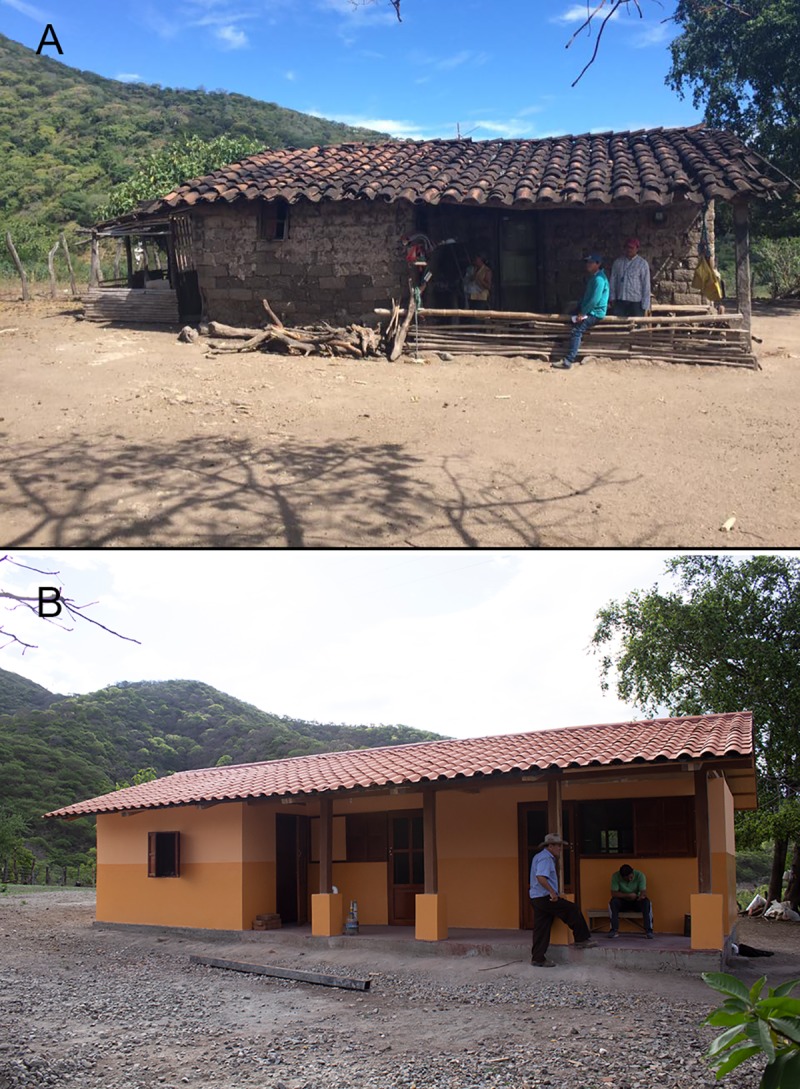
Exemplar of a HHHL home before (A) and after (B) intervention.

The cost of HHHL interventions ranged from US$2,000 to US$4,000 in refurbished homes, and US$15,000 to US$20,000 for fully reconstructed homes (Fig 1). Implementation was mostly funded through research schemes available at the Pontifical Catholic University of Ecuador and Ohio University; the remaining expense was covered with contributions from partner families themselves and institutional partners. Family contributions ranged from US$500 for refurbished homes to $2,500 for fully reconstructed homes. The National Ministry of Urban Development and Housing (MIDUVI) provided partial funding through existing housing subsidies for the construction of two of the intervened homes, and Gobierno Autónomo Descentralizado (GAD) del Cantón Calvas (local government) contributed machinery and technical personnel in different phases of the project. This model resembles other forms of community involvement and intersectoral collaborations previously implemented in Bolivia and Brazil [16,22].

### Study design

This study was part of a larger interdisciplinary research project conducted around HHHL using multiple methodologies and multiple sources of information, including entomological and epidemiological data. To analyze sustainability from a social perspective, we conducted a focused ethnographic study informed by grounded theory (GT). GT is a systematic approach to data collection and analysis based on emerging information grounded in actual data rather than in theory. Because of its capacity to collect rich data and gain deeper insights into participants’ experiences, GT is usually paired with ethnographic methods [51]. Practices common to the two methods applied in this study include simultaneous data collection and analysis; identification of emergent themes in early stages of data collection; inductive construction of categories aimed at explaining processes described in the data; and, integration of categories into a larger explanatory framework [52].

GT uses sensitizing concepts to guide the first steps into data collection [51]. In this case, we used sustainability as sensitizing concept. Multiple definitions have been used in implementation reasearch to address issues of sustainability. Some definitions emphasize elements of ownership and appropriation. The Ecohealth approach, for example, suggests that sustainable initiatives are those capable of effectively addressing local priorities, switching external perceptions and motivating wider economic, political, or even environmental changes [53]. More popular are definitions that refer to sustainability as a time-bounded concept; issues of maintenance and durability are central in this perspective [54]. Finally, systems’ thinking reframes sustainability as a fundamental characteristic of resilient systems. From this perspective, a system is sustainable because it can respond to the movements, changes, and behaviors of its constitutive elements and environment [55]. These three elements (ownership, temporality, and systemic responsiveness) were used as reference for data collection and analysis in this research. Additionally, sustainability was operationalized in interview protocols and questionnaires according to the Pan American Health Organization (PAHO) parameters for sustainable management of NTDs in Latin America [56,57]: (i) vector control; (ii) provision of water and sanitation; (iii) management of zoonotic elements of the disease; and (iv) community participation.

### Sampling

Participants were purposively selected from inhabitants of the intervened communities. To organize data collection and facilitate comparison in later stages of analysis, participants were divided into partner families and non-partner families. The first group included the six families that agreed to partner with HHHL to build or improve their homes. The second group included families (6) exposed to the intervention selected to closely match factors considered in the identification of partner families, specifically decay level of the homes and socio-economic conditions. These families were included to depict their understanding of health in relation to their homes and compare it with the information obtained from the previous group.

### Data collection

Using an ethnographic approach, we followed the negotiation and construction process with all the partner families between 2013 and 2016. Additional data were collected between 2016 and 2017 through in-depth interviews, participant observation, informal conversations and document review. All data were collected by authors CN-S and DG. Author DG worked as local facilitator of HHHL during the homes’ improvement process.

#### In-depth interviews

In-depth interviews [58] were conducted in Spanish, as this was respondents and interviewers’ first language. Thirty-six (n = 36) individuals in partner and non-partner families were interviewed in several opportunities before, during and after interventions (Table 4). All interviews were audio recorded.

**Table 4:** Interviewees in partner and non-partner families.

Case	Total Occupants	Village	Interviewees
Partner family 1	9	GA	Male (55), Female (56), Male (15)
Partner family 2	7	CH	Female (98), Female (38), Female (16)
Partner family 3	4	BM	Female (81), Male (48), Female (50)
Partner family 4	6	BM	Male (55), Female (56), Male (16), Female (18).
Partner family 5	5	GA	Male (54), Female (42), Female (77)
Partner family 6	5	CH	Male (44), Female (37), Female (17), Female (15)
Non-partner family 1	11	BM	Male (46), Female (42)
Non-partner family 2	6	BM	Male (30), Female (28)
Non-partner family 3	5	CH	Male (48), Female (49), Female (22)
Non-partner family 4	7	BM	Male (33), Female (29)
Non-partner family 5	4	GA	Male (71), Female (60)
Non-partner family 6	8	CH	Male (40), Female (42), Female (17)
Non-partner 7	6	BM	Male (36), Female (29)

*GA = Guara; CH = Chaquizhca; BM = Bellamaria

#### Participant observation and informal conversations

Participant observation and informal conversations [59] were conducted during the year following completion of homes’ interventions. Observation periods were organized during the dry and rainy season. Field notes were completed immediately after participant observation and informal conversations conducted by author CN-S.

#### Document review

Annual reports produced by HHHL were reviewed. These documents were used as secondary sources to guide the development of the interview guides [60] and to contribute to the immersion required to develop the ethnographic perspective proposed in this study. Documents reviewed included research reports submitted to funders in 2013, 2014, 2015 and 2016, as well as weekly reports between 2013 and 2016. As HHHL’s field coordinator, co-author DG was involved in the production of these reports.

### Data analysis

Data were analyzed in two phases: first, as an ongoing and iterative process taking place simultaneously with data collection [61] and once all data were collected and organized, as a comprehensive coding process [62]. Initial coding stages involved assigning codes to words or larger segments of transcribed materials; in-vivo and process coding were used as main approaches in this phase [62]. A total of 382 initial codes were identified and organized in Nvivo 11.4 software [63]. Coding schemes were constructed in Spanish using a line-by-line approach and memo-writing was used to synthesize, integrate, and organize initial codes before moving into the phase of focused coding [64]. Six main categories were identified for theory construction through a series of comparative exercises, including perspectives of partner and non-partner families, temporal references (before and after intervention) and differences between interventions. Finally, these categories were integrated into four concepts built during the phase of theoretical sorting as described in the results section [64,65].

### Ethical approval

Research protocols were approved by the Institutional Review Board at Ohio University (16-X-209) and the Research Ethics Committee at the Pontifical Catholic University of Ecuador (Oficio-CEISH-232-2016). Written informed consent was obtained from all participants. Written minors' assent and parents' informed consent were requested for participants under 18 years old.

## Results

We identified four levels of impact of the housing intervention: health, emotional, economic and social impacts.

### Perceived health impacts

Local families perceived that the HHHL’s model promoted improvements in four health-related areas: (i) safety; (ii) vector control; (iii) water, hygiene, and sanitation; and (iv) separation from animals.

#### Safety

A particular sense of urgency was expressed by members of the partner families as motivation to accept the idea of reconstructing their homes using the HHHL model. These participants did not refer to specific concerns regarding triatomines or disease presence; instead, safety of their families was mentioned as a determining factor for their decision:

My house couldn't resist more. I was very concerned thinking that it would

collapse during the next rainy season because the walls had profound cracks. I

had no option but immediately building a new house. *Male*, *48*.

Our previous house was about to kill us. Everything was poorly done, poorly built… it was moldering. *Female*, *56*.

Local families perceive damages in the roof as more serious than other structural problems, particularly during the rainy season. Constant rain, strong winds, and mudslides make the structural problems of traditional homes more pressing, as explained in the following quote:

What I like the most about this new house is that we don't have to deal with rain during the night. The previous house had so many leaks that we had to squeeze one against the other in our beds to avoid them. There is no dirt falling in our beds because water cannot bring it inside anymore. *Male*,*17*.

HHHL homes were generally perceived as safer also because of their structural features. HHHL homes were built with adobe blocks but using updated construction techniques designed to reduce their size and bring more stability to the construction. In order to slow down decay and avoid cracks, adobe walls were covered with mesh and plastered with cement. Participants expressed that walls’ steel rebar rods and concrete foundations are increasingly resistant to tremors and landslides. Similarly, the mechanically compressed adobes used in the construction were seen as of better quality than the traditional ones (manually made). Lastly, cement plastering was perceived as an efficient way of protecting the home from decay:

The walls of our previous house were cracking. We were trying to make some adobe to raise them again, but then we realized that the roof was also moldering. That does not happen with cement. *Female*, *37*.

#### Vector control

Partner families perceived an important reduction of insects—not only triatomines—within the new homes. As described by a head of household,

We still have some bugs coming from time to time, but you cannot compare with the previous house. We can rest at home assured that there are not even mosquitoes around. In the old house they didn't let us sleep during the rainy season. In addition to bugs, we had rats peeing on the beds or falling from the roof. We don't have any of that anymore. *Male*, *54*.

The HHHL model installed permanent mesh screens in windows and doors as ongoing protection against triatomines. Even though this element is not common in traditional constructions in this region, partner families showed high acceptability of this measure:

I feel comfortable and happy with my house because it is really fresh. I can even sleep with the windows wide open: the mesh protects us and nothing else is necessary. *Male*, *55*.

Multiple bugs were found around the mesh in windows of kitchens and rooms, showing their effectiveness to keep vectors out from internal spaces. However, we observed several quality issues in relation to the screens. Tears and openings were more common in rooms with frequent circulation of people. Participants identified the low quality of construction materials, strong winds, children introducing objects or pushing the doors from the mesh, installation problems, and faulty design as main causes for tears. In most cases, partner families found ways to repair the mesh when broken.

In addition to measures installed by HHHL, partner families continued applying traditional practices to protect their homes against insects, particularly insecticide spraying and turning off light bulbs so insects are not attracted. Partner families were able to establish connections between the perceveid reduction of bugs and heath protection:

For me, this is a new way of living. Our previous house was full of cracks, and bugs and animals could come very easily, but they don't come here anymore, they are always outside (…) [they] can transmit diseases to us, but since they are far away, we have a better way of living now. *Female*, *17*.

In contrast, non-partner families expressed several concerns in relation to the overall structure of their homes and its connection with disease occurrence:

I can protect my family from the cold and the wind with cloth rags or bed sheets. But I cannot do anything about diseases that come from flies, mosquitoes or *chinchorros* [local name for triatomines]. They come into our home because there are many cracks and we cannot cover them all. Even if I cover the cracks in the walls, they will come through the roof because we have tiles but not a ceiling. So, I cannot protect my family from them; bugs will always find a way to come in. *Male*, *46*.

#### Water, hygiene, and sanitation

All the interviewees reported having regular access to water at their homes, mainly through water systems built in the communities in the last ten years. Both partner and non-partner families expressed appreciation for having access to this resource to cover their cooking, hygiene, and animals’ watering needs at home.

The availability of water at home facilitated the construction of sanitary facilities—mainly latrines—in almost all homes in the communities. Although existing, they are usually not in use due to failures in the conduction system and outflow drains in the toilets located in the peridomestic area. Following cultural practices in this regard, sanitary facilities in the intervened homes were detached from the main house and located in the peridomestic area. HHHL intervention brought water to latrines and showers through hoses or pipes.

All partner families reported regular use of these facilities. However, the experience of lacking water at home and the need to secure access during the dry season has reinforced the practice of water storage in buckets and tanks attached to laundry sinks. This situation is especially visible in Bellamaria, where intermittent access to water has been a historical problem with devastating effects on productive activities.

In terms of hygiene, partner families stated that cleaning practices have not radically changed in their new houses, but that the results of their cleaning efforts are more visible now. Some of them acknowledged that they feel more motivated to clean in the new home:

I remember that my previous house was quite messy because we used to keep animals inside. My chickens were everywhere and since my kitchen was made out of bahareque [intertwined canes attached with mud], it did not look so clean (…) Cleaning and organizing take more of my time now, but I feel more comfortable. Unlike myself, I want my kids to grow in a clean place. *Female*, *38*.

Regular cleaning, as well as keeping animals and insects away, were seen as effective ways of keeping the house in good conditions. However, both activities demand ongoing work. Due to the dry nature of the terrain, there is ongoing circulation of dust that covers belongings and food. Members of non-partner families can appreciate easier cleaning as an advantage of the new homes:

Those [intervened] homes have the advantage of looking clean and organized (…) In houses like mine [traditional] you can sweep and sweep, but there is no way that you can keep them clean. *Female*, *60*.

Partners families stated that it is easier to clean the patio now that animals are not around because they do not have to deal with their feces and leftovers. However, peridomiciles still fulfill multiple productive needs, including storage of crops, daily food and tools, as well as corn and peanuts threshing, all of them producing additional amounts of detritus that local families have to dispose on regular basis.

#### Separation from animals

The practice of separating animal housing from the family’s space was constantly reinforced through HHHL’s health promotion activities. As explained by one of the partners:

One thing the program wanted us to do was living separated from chickens, preventing them from nesting in our rooms. We sent animals to sleep far away from the homes and now they are in their own place. *Male*, *44*.

Creating a physical separation between animals and families is a significant change in families’ routines. Traditional knowledge about animal husbandry suggests keeping chickens and guinea pigs in areas of the house where they can stay warm and ‘grow better,’ including the kitchen and under the beds. HHHL’s challenges to traditional practices have generated clashes and internal negotiations within families:

We have struggled a lot trying to convince my grandmother of sending animals away. She wants chickens to keep nesting in her bed. I have to be strong with her and argue because I don't want her to keep animals inside [the house], and even less so will I let them nest here. I gave up at the end and let her feed her chickens on the porch. She is old, almost 99, you know? She cannot walk much, so, I agreed. I feed my chickens outside the fence and she feeds hers here, in front of the house. *Female*, *38*.

HHHL’s interventions secured the construction of a fence that could function as permanent separation between animals and families. However, this separation is highly dynamic, as animals and produce circulate to and from the families’ plots on regular basis. For example, domestic animals look for a covered shelter more often during the rainy season; therefore, it was more common to see them around the porch and other roofed areas of the house during this period.

Community members value having a fence to separate animals—particularly pigs and goats—from activities conducted in the house. Families identified practical advantages derived from this practice as explained in the following quotes:

We didn't have a garden before because the patio was open and there were animals eating the seeds and the sprouting plants every time I tried. *Female*, *37*.

The benefits of this process are obvious; everybody in the house feels them. Fencing our land, for example, reduces the entrance of garbage and animals; because of that, we don't have to deal with their excrement and as a result, we are protected from diseases. *Male*, *55*.

Most partner families have adopted the practice of keeping animals away by feeding them and locating their food far from the main structure of the house:

I used to have a bucket full of leftovers for the pigs close to my house; animals were always around trying to get some food. It is not like that anymore because now we bring the leftovers up to the place where we keep our pigs. They do not come to the house at all. This is better because they used to defecate here, next to us. It is much better now because they eat, stay, and defecate in their own place. *Male*, *55*.

Guinea pigs are particularly challenging to keep outside the home. Traditional knowledge says that guinea pigs grow better in places where they can stay warm and in contact with firewood stoves. Partner families have created alternative structures outside the homes where they can replicate these traditional practices. Made out of rags and mesh, these structures provide an intermediate solution between the practice of keeping animals away from the home and families’ traditional knowledge.

Despite these benefits, the economic situation of the families impacts their decisions about the best way to keep their animals, as explained in the following quote:

Our pigs stay free most of the time because they can feed themselves with the grass they find around. Since we do not have enough money to buy food for them or to build a proper pigsty, our pigs only eat leftovers from what we grow and whatever they find around. If they depend on the food we can provide for them when they are locked in their corrals, they will be thin and we won’t be able to sell them. *Female*, *56*.

The fences built by the program have also shown quality issues. Some were built with fresh wood that expanded over time and wire rows did not stick properly to the wood. In other cases, animals ate part of the fence. Keeping fences in good shape can become a significant expense for the families, as they require generous amounts of wire to create multiple rows around the property that can effectively prevent animals from coming inside.

### Emotional impact

The second concept built in our analysis was emotional impact. Families expressed emotional impacts experienced along the processes of decision-making, construction and occupation of HHHL homes. The adjective most commonly used to describe the intervened homes, both by partner and non-partner families, was ‘beautiful.’ According to participants in this study, what makes HHHL homes beautiful is that ‘adobe is not exposed’, ‘they look clean’, ‘they are much better than previous homes’, ‘they are well done’, ‘it is a pleasure coming to visit’, and ‘they have more space, and everything looks more organized’, among others.

HHHL homes were perceived as aesthetically and technically superior to other homes in this area. This factor allows families to take pride on an asset they have actively achieved with their own effort. References to traditional homes in partner families compared past and present conditions, as illustrated in the following quotes:

I never thought I could have a house like this, but I got it through my own efforts. Of course, I had to work very hard, put my hands at work, but I cannot compare the kind of house I have now with the one I had before. This was built with all the strength I put into it. *Male*, *55*.

When asked about the most significant changes experienced in the new homes, families’ narratives included practical factors such as more capacity to receive visitors and to conduct activities at home, as well as more comprehensive ideas about the impact of the home on families’ lives. The following quotes illustrate this wide range of factors:

What has changed the most is that we don't have to live surrounded by dirt and dust now (…) Additionally, when our extended family comes to visit, we have space for them. The house is cleaner, more organized… my old house was not nice. *Male*, *17*.

In my opinion, what have changed the most are the bedrooms. We have more space now and I feel very happy because I can sleep separately with my female daughters now. The house is more organized. And I feel more comfortable because I have the bathroom right here… we don't have to go in the open air anymore. *Female*, *38*.

Partners’ comments connected factors such as hygiene, absence of animals, and access to water and sanitation with larger ideas about their wellbeing:

Everything has changed in our own health because there is a little bit of control, more cleanliness (…) Considering the extreme conditions we have faced, this is progress. It is pure happiness when I come from my plot and I can just sit anywhere without the concern of animals coming to bother me (…) My new granddaughter will not have to grow up with animals’ feces everywhere, unlike my kids that had to step on that dirtiness (…) I think I am good, things have improved, thanks God. *Male*, *55*.

Another common element in partner families’ narratives was the framing of the construction as a multiparty project in which they actively participated. Families believed that their own efforts were important to rebuilding their homes. This notion of effort is often linked with families’ ownership over the final product:

When people ask to me what I had to contribute for the project, I always tell them that I have to give all my work for the entire summer. Some of my neighbors really dislike that idea. But I tell them that for anything you want, you need to work. If there is someone who is offering to help you, that is even better, and you have to put even more effort into it. Nobody will give you everything for free; at the bare minimum, you need to work hard so you can appreciate what you have done. You have to make an effort, know that you have sweat for what you have, so you can also take care of it. *Male*, *55*.

Elders living with three of the partner families were more skeptical about the project and expressed resistance to changes in their living environments. In their opinion, the new constructions can affect their comfort and health in different ways. The following quote exemplifies their concerns:

This [dirt floor] is firm-land and my cane does not slip when I walk. Here I have all what I need (…) I do not want to go anywhere, and I prefer to finish my days here. If you can help me plastering my room with cement, fine, but do not do anything to the floor. I already know the holes of this dirt floor and do not want anything else. *Female*, *77*.

### Economic impact

The third concept that influences the sustainability of HHHL as a model was community members’ perceptions that although partnering with the project represented an important immediate cost for their finances, home improvement could have a positive impact on the economic situation of the family in the long run.

Even with the subsidies offered within the HHHL model (around 90% of the total cost), the expected contribution per family was significantly higher than traditional building costs (a traditional adobe house in this region can be completed at a total cost of US$1,000). Given their constrained economic situation, finding the necessary resources to carry out a project of this nature generated important economic demands as explained in the following quote:

Like most people around here, we are poor. We depend on our work, on what we can produce week by week, to sustain our family. We do not have capital or a regular salary. If we do not sweat on daily basis, who is going to give us a coin? Therefore, when we had to work for the house, we had to assume that nobody would get any income during the entire construction time. It was an entire summer thinking, ‘what am I going to live from?’ *Male*, *55*.

Even though partner families brought assets such as social capital and knowledge, all of them identified labor as the most important resource they contributed to the intervention. Partner families worked full time in the construction for periods ranging from 8 to 15 weeks during the most intensive period of agricultural production in the region (corn harvest). Additionally, some partners partially covered the costs associated with hired labor. While a traditional home can be built by two or three people in a month, HHHL homes required crews of minimum four people to be completed within two or three months. In these cases, partner families had to identify resources to pay one or two construction workers at a cost of US$10 to U$13 per day. Since heads of household can only engage in alternative economic activities when they are not attending their own plots, local families had to assume both the challenges of covering this cost and finding people available to work in the construction of the homes. This was particularly problematic during the rainy season, when local farmers rely on the labor of neighbors to collect their produce as fast as possible before it gets rotten. Besides these logistical considerations, the possibility of supporting community members with emerging jobs was mentioned as a positive impact of the HHHL model:

I was working in the construction of the homes when the construction of the water system in Bellamaria began. Since it was not possible for me to be in both places, I hired my brother in law to cover my part in [the construction] of the water system. That way, I was making some money and paying him—a little bit less—but we both had a job. *Male*, *30*.

Because of the perceived difference between the investment made by partner families and the quality of the final home, some community members, visitors and external contractors referred to the new homes as a ‘gift’. Families acknowledge that in economic terms, the contribution of HHHL was important and that even considering that some of them were saving resources for minor repairs or full reconstruction, the scale of HHHL intervention was much larger than what they initially envisioned. However, HHHL homes are seen as an investment towards the future as they reduce the need to attend to problems associated with the natural decay and structural damages of old homes:

I don't have to worry now about how to fix my house, how to buy more materials, or how to get the money to pay for more loans. That pain is gone, and my only concern now is how to get through life. *Female*, *56*.

Importantly, women’s participation in productive activities opened spaces for them to get involved in decision-making regarding the construction project. Traditionally, both men and women are involved in planting and harvesting, but women are the ones who take produce to the market. This responsibility gives them a prominent role as administrators of cash at home. As a consequence, even though most of the negotiations between HHHL and the families took place through the male head of household, women had a definitive word in the decision of joining the project:

I paid for most of the construction with my work. My husband helped in the adobe production and then in the construction; but most of the money we used to pay the people that helped here came from my work at the market, what we got from selling animals, and [additional] money that my older sons sent to us. *Female*, *56*.

Although economic constraints persist for partner families, new economic activities emerged with the new living environments. Four out of the six partner families have tried to organize a productive garden, while one of the families became involved in tilapia and poultry production in the surrounding areas of the house. Two of them have implemented basic drip irrigation systems in an attempt to sustain their production during the dry season.

### Social impact

This category expands the limits of the household level and connects HHHL implementation with larger social structures in the communities. Following existing dynamics of community organization, family members worked together closely in the construction of the model. Women and younger members of the family were in charge of cooking for the construction crews and organizing peridomestic areas. Additionally, they were involved in the production of adobe: unlike the traditional large and heavy blocks, the smaller size of the blocks used in the new homes facilitated this possibility. Considering that almost all community members were familiar with making adobe because of longstanding traditional practices, it was easy for families to use their preexisting knowledge to speed up the construction process. Family members also participated in the preparation of tiles and painting. One of the families got a door for the peridomestic area produced by one of their sons currently enrolled in high-school:

When the moment to complete the peridomicile came, my family had no money to buy a new door. Since I am taking a class in metallurgy at school, I asked my older brothers to buy the materials for me so I could build the door myself. I cut, took measures and welded. At the end I thought it was better because I put in practice what I already knew and learned more. At the beginning, it didn't come up well… but it was my first real job in metallurgy. *Male*, *17*.

Similarly, the construction generated additional employment opportunities for the communities in general. HHHL hired people previously certified in construction techniques by the National Professional Training Service (Servicio Nacional del Capacitación Profesional de Ecuador, SECAP). Organized by HLI in 2012, this training provided a base of knowledge in construction with clay that was then replicated through the HHHL model. People who followed this training have been prioritized (although not exclusively hired) for adobe production, truss assembly, and home construction. This cycle of learning and practice is explained by one of the trainees:

I have learned a lot from this project. Unlike other [community members] that didn’t even go to the [SECAP] classes, I’ve been practicing what we learned. It was not knowledge to keep on paper. I think that it was not interesting for some people, but it was interesting to me. I just missed one class and then I talked to the professor, caught up and passed the test (…) The only thing I cannot do in these homes is the floor because it needs precise knowledge. Other than that, I can do everything: I have made the adobe, raised walls, installed doors and windows, built roofs, and fixed kitchens. I can even read blueprints. *Male*, *30*.

In this context, an ongoing interplay between existing and specialized knowledge took place between local families, external contractors, and architects along the construction process. Local families valued their own knowledge and questioned the information provided by external actors:

One day all the partners talked among us. We said that people from the construction crew were organizing this process, but they didn't have as much experience with adobe production as we have. We have built all the homes in this region with adobe! One day a number of adobes turned into dust in our hands and we decided to do things differently. We knew that we were adding too much sand into the mix, so we modified the formula [given by the architects]. Then we showed the quality of those adobes to the construction crew and they agreed that our method was better. *Male*, *54*.

Similarly, partner families with more experience in construction provided opinions and advice regarding different phases of the process, such as the layout of foundations and roofs. Families’ previous involvement in the construction of traditional homes helped them to supervise the construction and guided them to consult with more experienced neighbors that had been involved in other projects. That knowledge remains with the families as part of the process:

I can tell you that if someone comes today to ask for help in the construction of this type of house, I would gladly provide it. If they want to make adobe, I can explain to them because I already know how to make it (…) I remember everything about the construction because I was there from beginning to end. I never abandoned the construction site. That’s why when they [construction workers] did something wrong, I could call them out (…) I have everything in my mind now. *Male*, *55*.

Having access to specialized knowledge was particularly valued given the steady lack of jobs in the region. Unemployment fosters ongoing migration of community members from rural villages to semi-urban areas with seasonal hiring of workers (mainly gold mines and shrimp production companies). Similarly, ongoing urbanization trends in Ecuador stimulate regular national and international outmigration. Although community members (mainly men) usually go to these places for short periods of time, the idea of permanent relocation in search for job opportunities is latent.

## Discussion

We hypothesise that sustainability of CD control under the model proposed by HHHL largely depends on the conceptualization of home improvement as a process that brings positive health outcomes, expressed not only as physical protection, but also as a wide range of emotional, economic, and social impacts on families’ wellbeing. The different levels of impact previously outlined—perceived health, emotional, economic and social—describe agency in individuals, families and communities to identify health protective practices and make decisions towards disease prevention in their own context. Considering the three characteristics of sustainability used to operacinalize this concept during data collection, i.e. ownership, temporality, and systemic responsiveness, we argue that sustainability of the HHHL model is enhanced by the confluence of three factors: systemic improvement of families’ quality of life, perceived usefulness of control measures, and flexibility to adapt to emerging dynamics of the context.

### Systemic improvement of families’ quality of life

The results previously presented show that systemic interventions around living environments have the potential to generate positive impacts in vector control, but also in aspects such as emotional and social wellbeing at the household and community levels [25]. Systemic perspectives are operated when families link infrastructure-related changes with more overarching concepts such as ‘progress’, and ‘happiness’. This wellbeing is also stated as positive evaluations of the future associated with a significant change in life conditions for younger members of the family. HHHL homes integrated ongoing interactions between local families and their natural environment in the construction of an idea of health that acknowledges the dynamics of rural life in the context of CD transmission in southern Ecuador. Instead of focusing exclusively on CD’ transmission dynamics, the model addressed areas perceived as more pressing by local families, including the urgency of living in a safe space, access to water, and the generation of job opportunities. All four kinds of impacts can be considered potential entry points to activate and maintain agency in relation to sustainability of CD prevention and health protection in the different actors linked to the microsystem of the home.

### Perceived usefulness of protective measures

Similar to previous interventions focused on infrastructure improvement [37,66,67], the use of local materials, reutilization of recycled parts of the old house, and deployment of passive construction and demolition techniques, facilitated the acceptability of the protective measures. In spite of previous associations of adobe with CD transmission [66–68], partner families reported a reduction of insects’ presence, as well perceived increased durability and comfort in HHHL homes. This efficiency cannot be isolated from other protective measures applied within the home space, particularly those operating in peridomiciliary areas; however, they show an important contribution of the technology implemented in this case. Similalrly, as it has occurred in other experiences of partial or full reconstruction [69,70], it cannot be claimed that these changes have been caused exclusively by the intervention in the infrastructure; however, they show an element of protection that is acknowledged by local families as beneficial. In this case, families showed active engagement with insect control measures, such as consistent use of screens in doors and windows (reported and observed in domiciliary visits), as well as reduction of accumulated materials in the peridomicile and separation from domestic animals. Changes in this aspect have been gradual and not free of conflict. Tensions over conflicting uses of the space, for example, were observed during post intervention visits, as different priorities (generational, productive, gender-specific, and otherwise) emerge in the process of negotiating traditional practices within a renovated home space. This issue should be carefully observed in future phases of the project. HHHL efforts to introduce local knowledge in the generation of protective practices may have reduced the impact of this change but have not eliminated the natural friction between traditional and introduced practices.

### Flexibility to adapt to emerging dynamics

The flexible design of the HHHL’s intervention facilitated the modification of the conditions of the program according to the resources and priorities available in each stage of the pilot phase [53]. This emergent response is an important attribute of system-based interventions: rather than providing a set of fixed answers, systems put resources in place to be able to identify disarrangement and modify interventions as they evolve. A system-based intervention of this nature facilitates the design of intermediate forms of implementation, flexible enough to incorporate existing and acquired knowledge and enhance decision-making power in local communities [71]. Consequently, and as shown across categories, families and local actors responded with creativity, resilience, and problem-solving capacity to complete the interventions proposed by the program. Sustaining this level of flexibility is fundamental to enhance participation of community members in order to sustain the benefits observed in this phase and bring the HHHL model up to scale.

Sustainability of the HHHL model can be hindered by multiple factors. Funding the implementation of the HHHL model is expensive for local families, even under the partnership model proposed by the project. Expansion of the HHHL model should consider the important levels of stress derived from the need to meet agreements made with the program and the lack of economic resources available to supply daily needs. Participants talked about their dependence on daily labor, as well as restrictions to get access to funding sources due to the unpredictability of their work as farmers. The economic cost of an intervention that demands ongoing contributions in labor and cash over a relatively long period of time is a factor that cannot be minimized.

Similarly, efficiency of construction materials should be revised considering environmental conditions such as extreme temperatures and profuse rains present in this area at different times of the year. Elements of the construction such as stoves, mesh, and wood have shown quality issues that demanded additional resources from partner families. Since the need for additional resources can act as an important barrier toward behavior change, addressing these quality issues is fundamental to enhance a long-lasting usage of protective measures.

A more detailed analysis of sustainability in refurbished homes is recommended. Partial interventions are challenged with structural problems of the original construction that could render the HHHL intervention irrelevant in the long run, including leaks and high internal temperatures.

Scaling up the HHHL model should further consider the internal composition and organization of the local communities. Internal homogeneity could have played a major role in facilitating the organization of activities, as well as participation of local families in different stages of the project. Most of the partner families had the classical structure of a father, a mother and a mix between young and older children permanently present in the household. This structure facilitated organization of labor, distribution of income generation activities and access to additional forms of funding. Single parent families and those with several children in school age experienced a greater burden when trying to implement the model, as they had fewer resources at hand. Demographic changes in relation to migration should also be carefully considered, as they can have an important impact on the final decision of investing resources to adopt the HHHL model in the long run.

Finally, reinforcing protective behaviors gains relevance as vector and disease become less visible [72]. Systematic and regular health promotion before, during and after the intervention is highly recommended to reinforce protective practices in partner families.

### Conclusion

Different from disease-centered approaches, systems thinking emphasizes that social structures are not just aggregates of parts: they are systems acting under concrete logics that produce and reproduce social orders through practices and relationships among actors. Human beings are not only pieces of these structures, but agents in their capacity to reconfigure their environment and the larger social groups they belong to through reflexive thinking articulated in discursive and practical action. In this context, the sustainability of the systemic health promotion model proposed by HHHL relies on its capacity to enhance the effectiveness of control strategies while directly improving the quality of life of individuals and communities at risk.

Although an independent study of cost-effectiveness study is recommended, our results suggest that the conditions of structural poverty experienced by local families are still the most important factor to consider when evaluating the viability of sustainably scaling up the HHHL model in this context. Contributions of partner families remained within the approximate US$250 to US$2500 (inflation rates adjusted) ranges previously calculated for infrastructure-based interventions implemented for CD control [73]; however, these contributions require significant commitments from already financially stressed local families. Intersectoral collaborations can provide alternatives for implementing the model within a system-based approach [18,53]. Since government institutions have already identified mechanisms to contribute to the implementation of the model (e.g., MIDUVI and GAD), seeking additional coordination with national organizations responsible for CD control in the country (Ministry of Public Health and National Service for the Control of Disease Transmitted by Arthropods, among others) is recommended. Partnering with private organizations with the experience and infrastructure necessary to implement home improvement projects at large scales can also be considered a way to avoid overpricing and accessibility issues experienced during this pilot phase of implementation. Upcoming publication of entomological results will constitute an important asset in funding allocation efforts.

### Limitations

Although participants for this research were selected on the basis of purposive and theoretical arguments, the small size of intervened homes limits our claims. Authors CN-S, DG, and MG have been engaged in HLI activities for an extended period of time; both participants’ answers and researchers’ analysis might be influenced by previous interactions during HHHL interventions. Authors SJ, EB, KP and BRB checked for potential biases during the analysis stages of the process. Future studies conducted to follow up performance and appropriation of the model will include additional interviewers involved over shorter periods of time. Lastly and in spite of the authors’ efforts to stay close the data, richness of local language and intentionality of the original words used by research participants might have gotten lost in translation.

## Supporting information

S1 Checklist COREQ checklist. (DOCX)
