# Supplementary material for: Home improvement and system-based health promotion for sustainable prevention of Chagas disease: A qualitative study
Source: PLoS Negl Trop Dis. 2019 Jun 13;13(6):e0007472. doi: 10.1371/journal.pntd.0007472 (PMC6592574; doi:10.1371/journal.pntd.0007472)
Supplement: S1 Checklist — (DOCX) [file pntd.0007472.s001.docx]

Consolidated criteria for reporting qualitative studies (COREQ): 32-item checklist

**System-based Health Promotion for Sustainable Prevention of Chagas Disease: A Qualitative study**

| **No** | **Item** | **Guide questions/description** |  |
| --- | --- | --- | --- |
| **Domain 1: Research team and reflexivity** | | | |
| Personal Characteristics | | | |
| 1. | Interviewer/  facilitator | Which author/s conducted the interview or focus group? | All data was collected by authors CN-S and DG. Reported in page 12. |
| 2. | Credentials | What were the researcher's credentials? *E.g. PhD, MD* | The researchers credentials include PhD, MA, and BA for CN-S and BA for DG. |
| 3. | Occupation | What was their occupation at the time of the study? | CN-S was a PhD candidate and DG was HHHL’s local facilitator. |
| 4. | Gender | Was the researcher male or female? | CN-S is female and DG is male. |
| 5. | Experience and training | What experience or training did the researcher have? | CN-S holds a PhD in Communication and Public Health. DG is Agricultural Engineer. Both authors were involved in multiple research projects and were involved in multiple qualitative research courses prior to this study. |
| Relationship with participants | | | |
| 6. | Relationship established | Was a relationship established prior to study commencement? | Authors CN-S, DG, and MG have been engaged in HLI activities for an extended period of time. Reported on page 30. |
| 7. | Participant knowledge of the interviewer | What did the participants know about the researcher? e*.g. personal goals, reasons for doing the research* | Participants were informed about the specific goals pursued with this research. Ethnographic approaches deployed for data collection facilitated ongoing interactions between researchers and local communities in which emerging questions were addressed. Participants were aware of authors CN-S, DG, and MG long term involvement with HHHL. |
| 8. | Interviewer characteristics | What characteristics were reported about the interviewer/facilitator? e.g. *Bias, assumptions, reasons and interests in the research topic* | Reported on page 30. |
| **Domain 2: study design** | | | |
| Theoretical framework | | | |
| 9. | Methodological orientation and Theory | What methodological orientation was stated to underpin the study? *e.g. grounded theory, discourse analysis, ethnography, phenomenology, content analysis* | We conducted a focused ethnography informed by grounded theory (GT). Reported in pages 10 and 11. |
| Participant selection | | | |
| 10. | Sampling | How were participants selected? *e.g. purposive, convenience, consecutive, snowball* | Participants were purposively selected from inhabitants of the intervened communities. Reported in page 11. |
| 11. | Method of approach | How were participants approached? e*.g. face-to-face, telephone, mail, email* | Participants were approached face-face. |
| 12. | Sample size | How many participants were in the study? | 36 individuals were interviewed multiple times before, during and after reconstruction. Reported in Table 4. |
| 13. | Non-participation | How many people refused to participate or dropped out? Reasons? | No refusals were reported. |
| Setting | | | |
| 14. | Setting of data collection | Where was the data collected? e*.g. home, clinic, workplace* | Data were collected at participants’ homes. |
| 15. | Presence of non-participants | Was anyone else present besides the participants and researchers? | Members of the same family were occasionally present during data collection activities. |
| 16. | Description of sample | What are the important characteristics of the sample? *e.g. demographic data, date* | Participant’s demographics were reported in Table 4. |
| Data collection | | | |
| 17. | Interview guide | Were questions, prompts, guides provided by the authors? Was it pilot tested? | Prompts for research questions were reported in page 11. |
| 18. | Repeat interviews | Were repeat interviews carried out? If yes, how many? | None. |
| 19. | Audio/visual recording | Did the research use audio or visual recording to collect the data? | All interviews were audio-recorded. Reported in page 11. |
| 20. | Field notes | Were field notes made during and/or after the interview or focus group? | Field notes were collected at all stages, but particularly after conducting observations and informal conversations. Reported in page 13. |
| 21. | Duration | What was the duration of the interviews or focus group? | Interviews lasted between 40 and 90 minutes. |
| 22. | Data saturation | Was data saturation discussed? | N/A |
| 23. | Transcripts returned | Were transcripts returned to participants for comment and/or correction? | No. |
| **Domain 3: analysis and findings** | | | |
| Data analysis | | | |
| 24. | Number of data coders | How many data coders coded the data? | Coding was conducted by CN-S under supervision of BRB. |
| 25. | Description of the coding tree | Did authors provide a description of the coding tree? | Coding structures were reported on page 13. |
| 26. | Derivation of themes | Were themes identified in advance or derived from the data? | Following the principles of Grounded Theory, themes identified were derived from the data. |
| 27. | Software | What software, if applicable, was used to manage the data? | N-Vivo 11.4 |
| 28. | Participant checking | Did participants provide feedback on the findings? | Partial findings were discussed with selected groups of participants. |
| Reporting | | | |
| 29. | Quotations presented | Were participant quotations presented to illustrate the themes / findings? Was each quotation identified? e*.g. participant number* | Yes, quotations were identified by gender and age. |
| 30. | Data and findings consistent | Was there consistency between the data presented and the findings? | Yes. |
| 31. | Clarity of major themes | Were major themes clearly presented in the findings? | Findings were extensively presented in the Results section (pages 14 to 25). |
| 32. | Clarity of minor themes | Is there a description of diverse cases or discussion of minor themes? | Findings were extensively presented in the Results section (pages 14 to 25). |
